# Proteomic Analysis in Diabetic Cardiomyopathy using Bioinformatics Approach

**DOI:** 10.4137/bbi.s313

**Published:** 2008-01-21

**Authors:** Allam Appa Rao, Hanuman Thota, Ramamurthy Adapala, Suresh Babu Changalasetty, Ramachandra Sridhar Gumpeny, Annapurna Akula, Lalitha Saroja Thota, Siva Reddy Challa, M.R. Narasinga Rao, Undurti N. Das

**Affiliations:** 1 Department of Computer science and Systems Engineering, Andhra University, Visakhapatnam-530003, India; 2 Department of Computer sciences and Engineering, Acharya Nagarjuna University, Guntur-522510, India; 3 Endocrine and Diabetes center,15-12-16 Krishnanagar, Visakhapatnam-530002, India; 4 Department of Pharmaceutical Sciences, Andhra University, Visakhapatnam-530003, India; 5 Department of Computer science, Annamailai University, Annamalai Nagar-608002, India; 6 UND Life Sciences, 13800 Fairhill Road, #321, Shaker Heights, OH 44120, U.S.A

**Keywords:** diabetes, cardiomyopathy, protein kinase C, calcium, metallomatrix proteins, advanced glycation end products

## Abstract

Diabetic cardiomyopathy is a distinct clinical entity that produces asymptomatic heart failure in diabetic patients without evidence of coronary artery disease and hypertension. Abnormalities in diabetic cardiomyopathy include: myocardial hypertrophy, impairment of contractile proteins, accumulation of extracellular matrix proteins, formation of advanced glycation end products, and decreased left ventricular compliance. These abnormalities lead to the most common clinical presentation of diabetic cardiomyopathy in the form of diastolic dysfunction.

We evaluated the role of various proteins that are likely to be involved in diabetic cardiomyopathy by employing multiple sequence alignment using ClustalW tool and constructed a Phylogenetic tree using functional protein sequences extracted from NCBI. Phylogenetic tree was constructed using Neighbour—Joining Algorithm in bioinformatics approach. These results suggest a causal relationship between altered calcium homeostasis and diabetic cardiomyopathy that implies that efforts directed to normalize calcium homeostasis could form a novel therapeutic approach.

## Introduction

Patients with diabetes mellitus are at increased risk of cardiovascular mortality (1). Diabetic cardiomyopathy is a distinct clinical entity that produces asymptomatic heart failure without evidence of coronary artery disease (CAD) and hypertension and manifests itself as diastolic dysfunction that could eventually lead to left ventricular hypertrophy and failure. Although diabetic cardiomyopathy entity has been well described for quite sometime, its precise molecular basis is still debated. Some of the pathological abnormalities described in diabetic cardiomyopathy are: presence of myocardial hypertrophy, impairment of contractile proteins, accumulation of extracellular matrix proteins, formation of advanced glycation end products, and decreased left ventricular compliance (2). Majority, if not all, of these abnormalities could be attributed to defects in the regulation of calcium homeostasis. In view of this, in the present bioinformatics approach we tried to identify key functional proteins that are closely associated with diabetic cardiomyopathy.

## Materials and Methods

We have collected those proteins that are believed to be involved in the pathogenesis of diabetic cardiomyopathy based on literature survey and reports. For instance, both Na^+^-Ca^2+^- exchanger and Na^+^-K^+^-ATPase are considered as the key proteins that are closely associated with diabetic cardiomyopathy (2, 3). Similarly, glucose transporter-4 (GLUT-4), MMP-2 (matrix metalloproteinase-2), protein kinase C (PKC), p38 mitogen-activated protein kinase, and CD36 are thought to play a significant role in diabetic cardiomyopathy. Hence, these proteins were selected for the present study (see Table 1). The functional protein sequences in FASTA format for these proteins were collected from NCBI (National Center for Biotechnology Information) (http\\www.ncbi.nih.nlm.gov). These sequences were given to ClustalW (http\\www.ebi.ac.uk\clustalw) for the Multiple Sequence Alignment. (which calculates the best match for the selected sequences, and lines them up so that the identities, similarities and differences can be seen). Based on these results, the scores table and phylogenetic tree that shows the distance between the selected protein sequences was constructed.

## Results and Discussion

**Figure 1:**
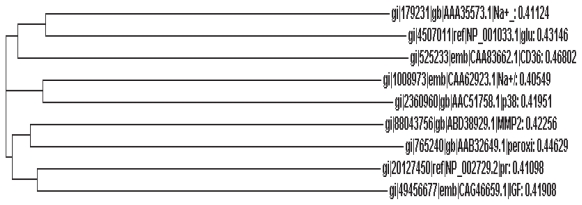
Phylogenetic tree that was constructed based on the alignment scores of all the protein sequences involved in diabetic cardiomyopathy. Proteins with minimum distance are Na^+^-Ca^+2^-exchanger, protein kinase C-β isomer and Na^+^-K^+^-ATPase.

The results of the present bioinformatics analysis given in Figure 1 showed that Na^+^-Ca^2+^- exchanger, protein kinase C-β, and Na^+^-K^+^-ATPase as the key proteins that could have a causal role in diabetic cardiomyopathy. This is based in the present observation that when phylogenetic tree was constructed based on the alignment scores of all the protein sequences selected showed that proteins with minimum distance are Na^+^-Ca^+2^-exchanger, protein kinase C-β isomer and Na^+^-K^+^-ATPase. This suggests that these proteins are not only closely linked to each other but also play a significant role in the pathobiology of diabetic cardiomyopathy. Both Na^+^-Ca^+2^-exchanger and Na^+^-K^+^-ATPase are involved in action potential generation and myocardial contractile function. Hence, any abnormality in their function could lead to myocardial dysfunction.

Long standing diabetes could produce myocardial dysfunction due to abnormalities in Na^+^, K^+^, and Ca^2+^ influx and efflux, in part, due to impaired Na^+^-Ca^+2^-exchanger and Na^+^-K^+^-ATPase activity. Such impairment in Na^+^-Ca^+2^-exchanger and Na^+^-K^+^-ATPase has the potential to cause abnormalities in myocardial function especially in the form of impaired diastolic relaxation and systolic contraction. These abnormalities could occur even in the absence of CAD and hypertension (3). The most typical feature of diabetic cardiomyopathy is the abnormal filling pattern of left ventricle with reduced compliance or prolonged relaxation (4). The shift in the faster V_1_ form of myosin to slower V_3_ form leads to delayed relaxation of the ventricles. Predominance of myosin subtype V_3_ reduces the Ca^2+^-ATPase activity (5). Impaired sarcoplasmic Na^+^-Ca^2+^-exchanger activity and depressed Na^+^-K^+^-ATPase activity causes retention of calcium that could render myocardial contractile dysfunction.

In addition, diabetic cardiomyopathy may be associated with aberrations in glucose and lipid metabolism (6), which are known to occur in patients with diabetes mellitus, that could lead to secondary disturbances in carbohydrate, lipid and adenine nucleotide metabolism in the diabetic heart (2). In particular, depletion of glucose transporter-4 (GLUT-4), increase in fatty acids, changes in calcium homeostasis, and associated small vessel disease, cardiac autonomic neuropathy, and insulin resistance may all play a significant role in the onset of diabetic cardiomyopathy.

It was reported that decreased sarcoplasmic reticulum Ca^2+^-ATPase activity results in decreased calcium transport in isolated sarcoplasmic reticulum obtained from animals and humans with diabetes mellitus. Furthermore, other abnormalities of Ca^2+^ homeostasis that could occur in these patients include: i) decreased Ca^+2^ uptake, ii) decreased Ca^2+^ binding, and iii) decreased Na^+^-K^+^-ATPase activity of sarcolemma (2). This is supported by the observation that sarcolemmal and the sarcoplasmic reticular calcium transporters are depressed in diabetic cardiomyopathy. This results in an increase in cytoplasmic calcium content and a decrease in calcium outward current resulting in prolonged action potential duration and increased myocardial stiffness. This altered intracellular calcium handling eventually leads to decreased myocardial function and failure (3).

Transgenic mice overexpressing PKC-β isoform developed cardiac hypertrophy and myocardial fibrosis, whereas PKC-β isoform inhibitor prevented several of the functional abnormalities seen in diabetic cardiomyopathy (7). This is supported by the observation that activation of PKC can modulate the gene expression of the myocardium that results in myocardial hypertrophy and myocardial fibrosis and eventually causes myocardial failure (8). Since PKC appears to be involved in the pathobiology of development of several complications seen in diabetes mellitus and as diabetic state itself induces the activation of PKC-β isoform that can produce cardiac abnormalities (as evidenced from the studies done with Transgenic mice overexpressing PKC-β isoform), the involvement of PKC in diabetic cardiomyopathy appears persuasive.

It is known that accumulation of myocardial collagen resulting in interstitial and perivascular fibrosis that occurs in long-standing diabetes can be correlated with early diastolic and systolic left ventricular dysfunction (9). Non-enzymatic glycosylation of collagen and diminished activity of collagen degrading enzymes such as matrix metalloproteinases (MMP) are considered to be pathognomonic of myocardial fibrosis (9) that could eventually lead to myocardial dysfunction in diabetes. There is also general consensus that formation of advanced glycation end products that occurs as a result of persistent hyperglycemia can also cause cellular and myocardial dysfunction in diabetes (10, 11). In addition, there is evidence to suggest that activation of the nuclear enzyme poly(ADP-ribose) polymerase (PARP) contributes to the development of endothelial and myocardial dysfunction in diabetes. It was reported that hyper-glycemia, cardiac PARP activation, a selective loss of endothelium-dependent vasodilation in the thoracic aorta, and an early diastolic dysfunction of the heart accompanied development of diabetes in experimental animals (12, 13). Treatment with PARP inhibitor, starting 1 week after the onset of diabetes, restored normal vascular responsiveness and significantly improved cardiac dysfunction, despite the persistence of severe hyperglycemia. The beneficial effect of PARP inhibition persisted even after several weeks of discontinuation of the treatment. Thus, PARP activation plays a central role in the pathogenesis of diabetic cardiovascular (cardiac as well as endothelial) dysfunction and PARP inhibitors may exert beneficial effects against the development of cardiovascular complications in diabetes.

It is evident from the preceding discussion that several pathological processes are proposed and seems to be involved in the development of diabetic cardiomyopathy. Of all these mechanisms, the earliest pathological event appears to be altered calcium homeostasis. The results of the present bioinformatics study also support the role of PKC in the development of cardiac abnormalities seen in diabetic cardiomyopathy. Thus, our bioinformatics study highlights the involvement of deranged calcium homeostasis and impairment of contractile proteins as the major responsible for the development of diabetic cardiomyopathy.

## Figures and Tables

**Table 1:** Table showing the genes/proteins that have been selected for the current study that are thought to be involved in diabetic cardiomyopathy as evident from literature survey.

S.No.	Gene name	Ac.No.	Length (amino acids)	Tissue type	Reference
1	Na^+^-K^+^- ATPase alpha-subunit	AAA35573.1	89 aa	Placenta, brain	14
2	Na^+^/Ca^2+^ exchanger	CAA62923	40 aa	airway smooth muscle	14
3	GLUT-4 transporter	NP_001033	509 aa	NOT SPECIFIED	15
4	MMP2	ABD38929	61	NOT SPECIFIED	16
5	Protein kinase C, beta isoform 2	NP_002729	673	NOT SPECIFIED	7
6	PPAR alpha	AAB32649	468	NOT SPECIFIED	17
7	p38 mitogen-activated protein kinase	AAC51758	365	NOT SPECIFIED	18
8	CD36	CAA83662	472		19
